# Activated IL-23/IL-17 pathway closely correlates with increased Foxp3 expression in livers of chronic hepatitis B patients

**DOI:** 10.1186/1471-2172-12-25

**Published:** 2011-04-14

**Authors:** Qinghong Wang, Yanhua Zheng, Zemin Huang, Yi Tian, Jijun Zhou, Qing Mao, Yuzhang Wu, Bing Ni

**Affiliations:** 1 Ministry of Education Key Laboratory of Child Development and Disorders, Pediatric Research Institute, Children's Hospital of Chongqing Medical University, Chongqing 400014, PR China; 2 Institute of Immunology, PLA, Third Military Medical University, Chongqing 400038, PR China; 3 Department of Infectious Diseases, Southwestern Hospital, Third Military Medical University, Chongqing 400038, PR China

**Keywords:** Treg, Foxp3, Th17, IL-23, IL-17, Hepatitis B

## Abstract

**Background:**

Foxp3 protein plays a critical role in mediating the inflammatory response and can inhibit the proinflammatory IL-23/IL-17 pathway. However, the molecular interplay of Foxp3 and the IL-23/IL-17 pathway in patients with chronic hepatitis B (CHB) remains unclear. To this end, we analyzed the expression patterns of Foxp3- and IL-23/IL-17 pathway-related proinflammatory cytokines in 39 patients with acute-on-chronic liver failure, 71 patients with CHB and 32 healthy controls.

**Results:**

Foxp3 expression was found to be elevated in and mainly expressed by the CD4^+ ^T cell sub-population of peripheral blood mononuclear cells and liver tissues of patients with hepatitis B. The intrahepatic expression of Foxp3 strongly correlated with the copies of HBV DNA and the concentration of surface antigen, HBsAg. IL-23/IL-17 pathway-related proinflammatory cytokines were also found to be significantly increased in patients' liver tissues, as compared to healthy controls. Moreover, Foxp3 expression was strikingly correlated with the production of these cytokines in liver tissues of CHB patients.

**Conclusions:**

The closely-correlated increase of Foxp3 and IL-23/IL-17 pathway activity in HBV-infected livers suggests that the proinflammatory IL-23/IL-17 pathway had not been effectively suppressed by the host immune machinery, such as Treg (Foxp3) cells. Constitutive activation of the IL-23/17 pathway, thus, may support the chronic hepatitis B state.

## Background

Hepatitis B virus (HBV) is a noncytopathic, hepatotropic DNA virus that is capable of inducing acute and chronic necroinflammatory liver injury [1]. Outcome of patients infected with HBV is closely associated with the extent of host immune response [2]. Moderate immune responses will mediate the clearance of HBV infection, while excessive responses will induce liver injury and low level responses will allow HBV residence. Unfortunately, patients with chronic hepatitis B (CHB) often exhibit impaired HBV-specific T cell responses [3]. The exact mechanisms of the immune system that fail in CHB infections remain unclear and, thus, remain a hindrance to the development of effective therapies.

Many mechanisms have been hypothesized to explain the inadequate immune responses that facilitate chronic HBV infection. The most recent has focused on the dysfunction of regulatory T (Treg) cells. Treg cells are characterized by their cell-specific expression of Forkhead box P3 (Foxp3) [4], a transcription factor that contributes to autoimmune functions. Studies have demonstrated that Foxp3^+ ^Treg cells can inhibit activation, proliferation and effector functions of several kinds of other immune cells, including CD4^+ ^and CD8^+ ^T cells, natural killer (NK) and NKT cells, B cells and dendritic cells (DC). As such, Foxp3^+ ^Treg cells are widely considered to be the principal mediator of dominant self-tolerance and immune homeostasis and to contribute to prevention of autoimmune disease, immunopathology and allergy [5]. In HBV patients, Foxp3^+ ^Treg cells have been associated with the incidence and extent of liver damage [6-10].

Chronic inflammation of the liver, known as hepatitis, is most commonly caused by infection with HBV. Many other inflammation-related diseases, such as multiple sclerosis, psoriasis, rheumatoid arthritis, inflammatory bowel disease, and asthma [5,11,12], have been characterized as having significant dysfunctions in the interleukin (IL)-23/IL-17 signaling pathway. Interestingly, IL-17 has been associated with the pathogenesis of chronic hepatitis B in humans [13,14]; however, the relationship between Foxp3^+ ^Tregs and the IL-23/IL-17 pathway (via Th17 cells) has not yet been investigated in HBV infected individuals.

This study was designed to elucidate the Foxp3-mediated activity of the IL-23/IL-17 signaling pathway in HBV-infected patients. We found that both Foxp3 and IL-23/IL-17 pathway-related cytokines were sharply increased in patients with CHB, as compared to healthy controls. We also determined that Foxp3 gene expression was positively associated with the presence of IL-23/IL-17 pathway cytokines at sites of inflammation. These findings suggest that both Foxp3 and the IL-23/IL-17 pathway are involved in inflammatory response to hepatitis B infection, and may interact with one another in CHB patients.

## Methods

### Patients and samples

One hundred and ten HBV-infected patients including thirty-nine acute-on-chronic liver failure (ACLF) and seventy-one chronic hepatitis B patients (CHB) were recruited for the study and enrolled when written, informed consent was obtained. Each patient was hospitalized and clinically followed upon release in the Department of Infectious Diseases, Southwest Hospital, Third Military Medical University. The study period lasted from December 2007 to January 2010. All individuals were confirmed as HBV positive, and negative for C and D types (HCV, HDV) and human immunodeficiency virus. Clinical records and self-reporting indicated that no anti-HBV agents or steroids were administered six months before study sampling. Twenty-four healthy blood donors (healthy controls, HC) and eight healthy liver tissue samples from liver transplantation patients were also selected. The study was approved by the ethics committee of the Third Military Medical University, Chongqing, China. Informed consent was obtained from all patients. The clinical characteristics of our patient cohort and healthy controls are shown in Table 1.

**Table 1:** Clinical characteristics of the study's subjects

Subjects	Cases	Sex (F/M)	Age in years (mean, range)	ALT in U/L (mean, range)	HBsAg^+^	HBeAg^+^	HBcAb^+^	HBV DNA^+^
HC	32	4/14	27 (17-42)	< 40	0	0	0	0
CHB	71	8/63	32 (18-42)	136 (53-326)	71	71	71	71
ACLF	39	3/36	28 (20-39)	206.34 (92-1675)	39	9/39	39	39

### Flow cytometry (FCM) analysis

The following antibodies were used: AF750-conjugated anti-CD3 (eBioscience, San Diego, CA, USA), PerCP-Cy5.5conjugated anti-CD4, and APC-conjugated anti- Foxp3. Peripheral blood mononuclear cells (PBMC) were obtained by Ficoll gradient. Cells were then stained to detect relevant surface markers, fixed, permeabilized with Cytofix/Cytoperm solution (BD Biosciences), and stained with anti- Foxp3. Detection was carried out on a FACSAria cell sorter (BD Biosciences), and data were analyzed by FlowJo v. 6.1 (TreeStar, Inc., Ashland, OR, USA).

### Total RNA isolation and quantitative PCR

PBMC and liver tissues were dissolved in TRIzol (Invitrogen, Carlsbad, CA, USA) for total RNA extraction. RT-PCR was carried out on the RNA product by using the PrimeScript™ RT reagents kit (TaKaRa, Shiga, Japan), following the manufacturer's directions. Quantitative real-time (q)PCR was performed using the SYBR^® ^Premix Ex Taq™ (TaKaRa). Gene-specific primers for human IL-17, IL-23, Foxp3, TNF-α and IL-8 were designed as shown in Table 2. Fluorescence signals were measured after 40 PCR cycles, and all samples were normalized to GAPDH housekeeping gene levels. All samples were run in duplicate.

**Table 2:** Primer design for detection of target genes

Genes	Forward primer	Reverse primer
Foxp3	5'-AAGGAAAGGAGGATGGACG-3'	5'-CAGGCAAGACAGTGGAAACC-3'
IL-23	5'-GCTTCAAAATCCTTCGCAG-3'	5'-TATCTGAGTGCCATCCTTGAG-3'
IL-17	5'-TCAACCCGATTGTCCACCAT-3'	5'-GAGTTTAGTCCGAAATGAGGCTG-3'
IL-8	5'-TTCTAGGACAAGAGCCAGGAAG-3'	5'-GGGTGGAAAGGTTTGGAGTATG-3'
TNF-α	5'-ATGAGCACTGAAAGCATGATCC-3'	5'-GAGGGCTGATTAGAGAGAGGTC-3'

### Immunohistochemistry analysis

Liver tissues were embedded in paraffin using the standard protocol. Immunohistochemistry for human Foxp3 was performed as previously described, with minor modifications [9]. Briefly, masked antigens were uncovered by microwaving for 20 min in citrate buffer, pH 6.0. After cooling at room temperature, non-specific binding was blocked by exposure to 5% bovine serum albumin (BSA) in phosphate-buffered saline (PBS) for 30 min. Incubation with primary antibodies for Foxp3 (Abcam, Cambridge, MA, USA) was carried out for 48 h at 4°C in a humidified chamber before the slides were incubated with secondary, biotin-conjugated antibody, followed by sequential incubation with horseradish peroxidase-streptavidin and the peroxidase substrate 3'-diaminobenzidine. Samples were then counterstained with hematoxylin.

For the double staining of anti- Foxp3 plus anti-CD4 and anti- Foxp3 plus anti-CD8, the first antibody (diluted 1:200) was applied for 48 h at 4°C in a humidified chamber. The second antibody, anti-human CD4 and CD8 (diluted 1:100 in PBS containing 5% BSA; eBioscience), was then applied for overnight at 4°C. CY3-labeled anti-goat IgG (diluted 1:500 in PBS) and Dylight488-labeled anti-mouse IgG (diluted 1:200) (Zhongshan Goldenbridge Biotechnology, Beijing, China) were applied for 60 min at 37°C. Nuclear counterstaining was performed by incubating stained samples with diaminidophenylindol (DAPI, 1:100 in PBS; D1306, Invitrogen) for 5 min. Normal mouse or goat IgG was used as a negative control. Images were obtained by the digital confocal laser scanning system MRC-600 (Bio-Rad, Hercules, CA, USA).

### Western blot

Total protein of liver tissue was extracted using T-PER^® ^Tissue Protein Extraction Reagent (Pierce, Rockford, IL, USA) and quantified with BCA™ Protein Assay (Pierce). The proteins were separated on a 16% SDS-PAGE and transferred onto polyvinylidene fluoride (PVDF) membranes for immunoblotting. The membranes were incubated with goat anti-human IL-17 (R&D Systems, Wiesbaden, Germany), mouse anti-human IL-23p19 (BioLegend, San Diego, CA, USA) and mouse anti-human Foxp3 (eBioscience, San Diego, CA, USA) for 1 h at room temperature, respectively. After incubation with peroxidase-conjugated rabbit anti-goat IgG, or rat anti-mouse IgG for 1 h at room temperature, specific protein bands on the membranes were visualized by the enhanced chemiluminescence method (Amersham, Piscataway, NJ, USA), according to the manufacturer's instructions.

### Virological assessment

The virological assay was performed as previously described [15].

### Statistical analysis

Comparisons between various groups were performed using the Mann-Whitney *U *test. Results are expressed as the mean ± standard deviation (SD), unless otherwise noted. Correlations between variables were evaluated using the Spearman's rank correlation. For all tests, 2-sided *P *< 0.05 was considered significant.

## Results

### Foxp3 expression is significantly high in peripheral blood of CHB patients

**Figure 1:**
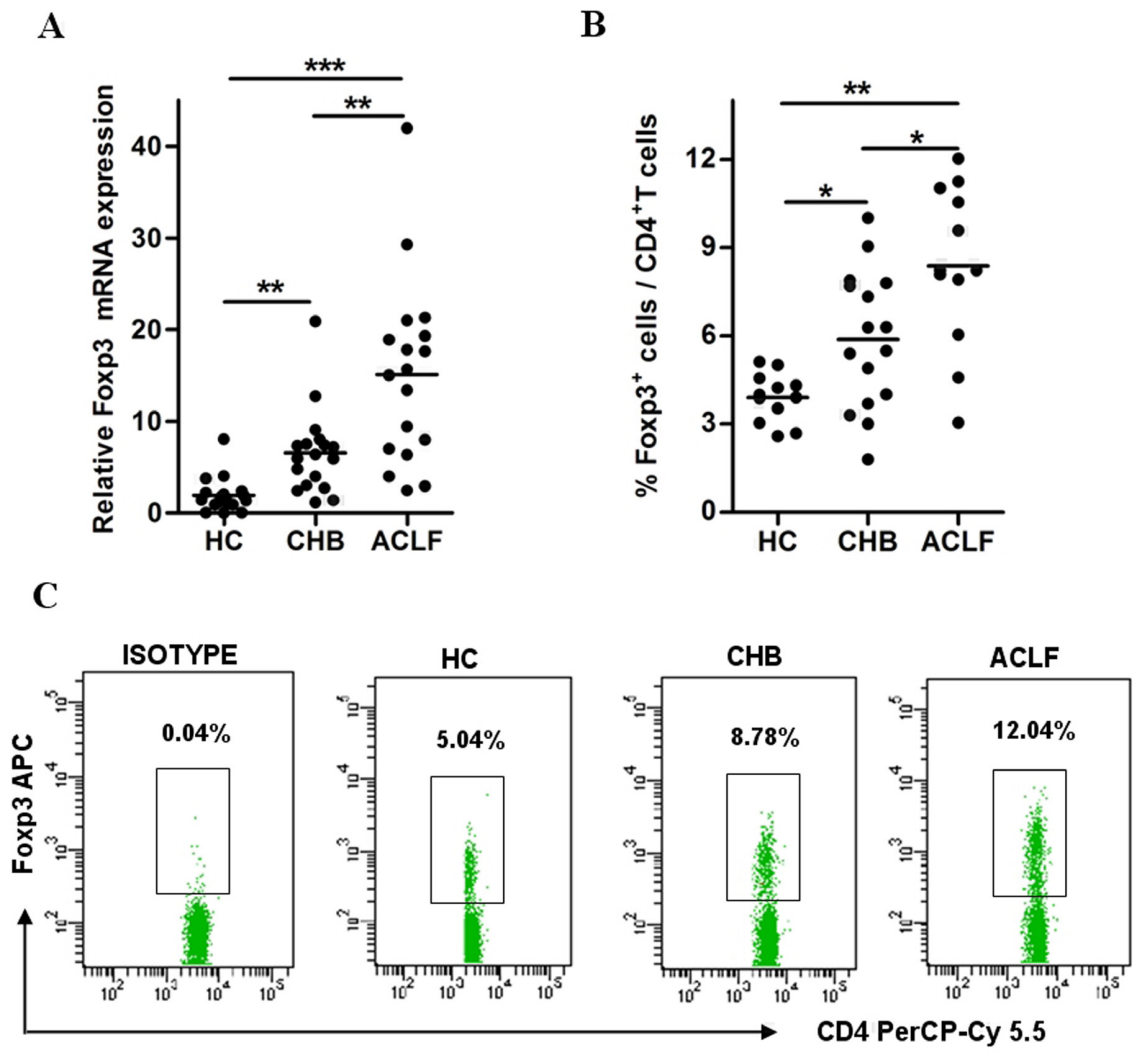
**Foxp3 expression is significantly higher in peripheral blood of patients with CHB**. PBMC from healthy controls and patients with CHB or ACLF were used to detect the expression levels of Foxp3 by qPCR (**A**) and flow cytometry (**B **and **C**). (**A**) Relative mRNA expression of Foxp3 in PBMC. (**B**) Pooled data indicating the percentages of Foxp3^+ ^cells in total CD4^+ ^T cells. (**C**) Representative dotplots of Foxp3^+ ^cells in CD4^+ ^T cells. The values in the quadrants indicate the percentage of each CD4^+ ^T cell subset. Error bars indicate SD; *, *P *< 0.05; **, *P *< 0.01; ***, *P *< 0.001.

Tregs with transcription factor Foxp3 play an pivotal role in controlling immune response mediated inflammation [16]. Several reports have shown evidence that Treg cells are involved in hepatitis B pathogenesis [6-10]. To further explore the role of Foxp3 in patients with hepatitis B, we collected the peripheral blood from 34 CHB patients and 30 ACLF patients, and then evaluated the expression of Foxp3 as detected by qPCR and FCM assays. As shown in Figure 1A, the expression of Foxp3 mRNA was found to be significantly increased in PBMC of CHB and ACLF patients, as compared with healthy controls. FCM assays indicated that the Foxp3^+ ^T cell portion of CD4^+ ^T cells were markedly up-regulated in HBV-infected patients; the ACLF patients had the most Foxp3^+ ^T cells (Figure 1B and 1C).

### Foxp3 is significantly augmented in liver tissue of patients infected with HBV

**Figure 2:**
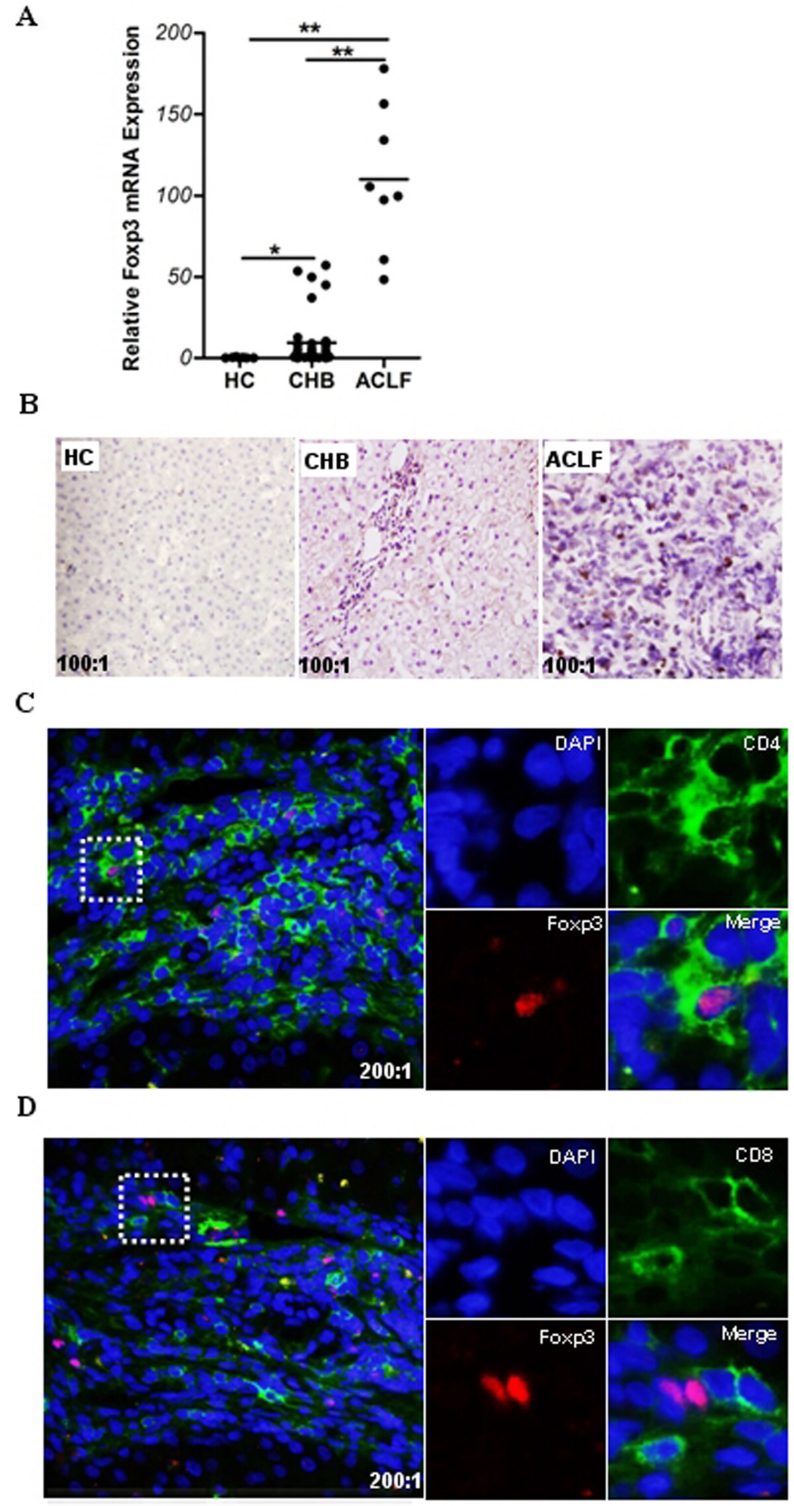
**Foxp3 is significantly augmented in liver tissue of patients infected with HBV**. Liver biopsy samples from healthy controls and patients with CHB or ACLF were used for the detection of Foxp3 mRNA by qPCR (**A**) and visualization of Foxp3 by immunohistochemistry (**B**) and laser scanning confocal microscopy (**C**,** D**). (**A**) Relative mRNA expression of Foxp3 in liver tissues. Error bars indicate SD; *, *P *< 0.05; **, *P *< 0.01. (**B**) *In situ *expression of Foxp3 in liver tissues. The Foxp3 expressing cells are stained brown. Representative results are shown from three independent experiments (100 × magnification). (**C**, **D**) Co-localization of Foxp3 with CD4 or CD8 in liver tissues from CHB patients. Anti- Foxp3 (red); anti- CD4 (green); anti- CD8 (green); and DAPI (blue). Representative results are shown from three independent experiments (200 × magnification). The right panels represent the enlarged portion of the indicated (white hatched box) area in the left panels.

To further value the role of Foxp3 in patients with hepatitis B, we detected the expression level of Foxp3 in liver tissue from patients infected by HBV by using qPCR and immunohistochemistry techniques. Concordant with the data from Xu *et al. *[9] and Janssen's group [8], Foxp3 was found to be mainly localized to cells near the hepatic portal tract, and Foxp3 expression was significantly higher in HBV patients than in healthy controls (Figure 2A and 2B). We further investigated the distribution patterns of Foxp3 expression in HBV-infected liver tissue by immunofluorescent confocal microscopy. Results revealed that Foxp3 in HBV-infected liver tissues was predominantly present in CD4^+ ^T cells, and not in CD8^+ ^T cells (Figure 2C and 2D).

### Increased Foxp3 expression is positively correlated with HBV persistence in host liver

**Figure 3:**
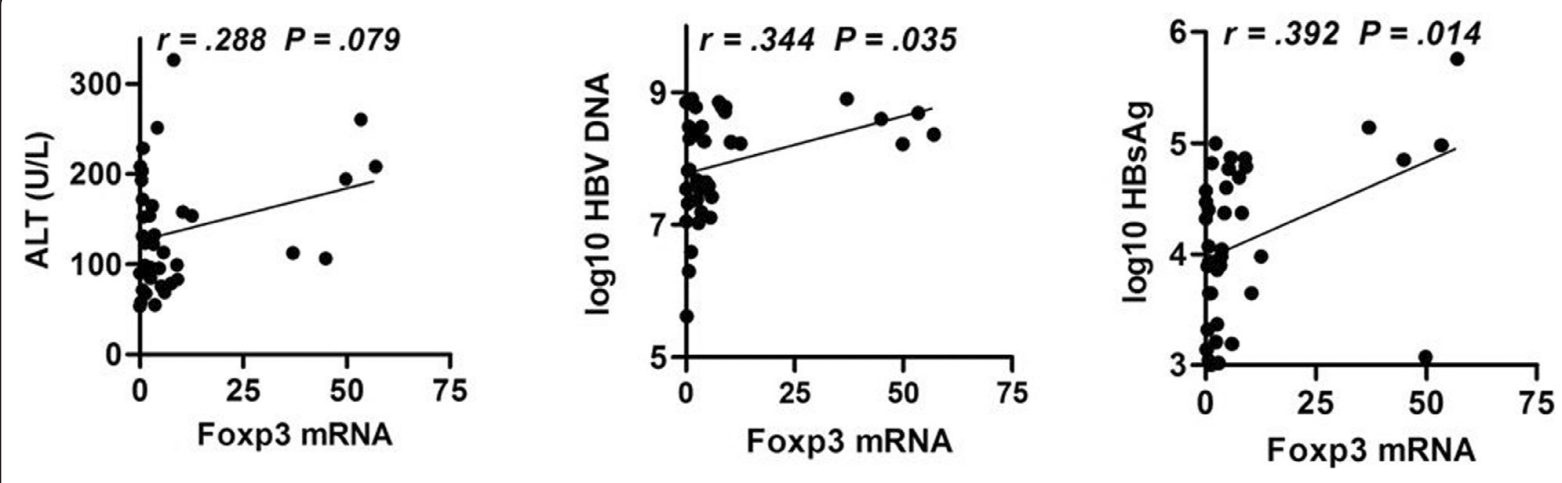
**Increased Foxp3 expressions is positively correlated with HBV persistence in host liver**. Liver biopsy samples from 38 CHB patients were used for the detection of Foxp3 mRNA by qPCR. Sera and plasma samples from CHB patients were measured for ALT level, log10 HBsAg (IU/ml), or log10 HBV loads (copies/ml). The relationships between Foxp3 and clinical symptoms of these patients were analyzed. *P *< 0.05 was considered significant.

In order to evaluate the relationship between Foxp3 expression and clinical symptom of patients with CHB, we analyzed the Foxp3 expression in liver tissue and plasma HBV DNA copies, hepatitis B surface antigen (HBsAg) concentration, and serum alanine aminotransferase (ALT) level in 38 CHB patients. There was significant association between Foxp3 expression and HBV DNA copies or HBsAg concentration, but not ALT (Figure 3). These results suggested that Foxp3 was involved in the persistent presence of HBV in CHB patients, but not directly involved in liver injury.

### Expression of IL-23/IL-17 pathway proinflammatory cytokines was significantly high in HBV-infected liver tissues

**Figure 4:**
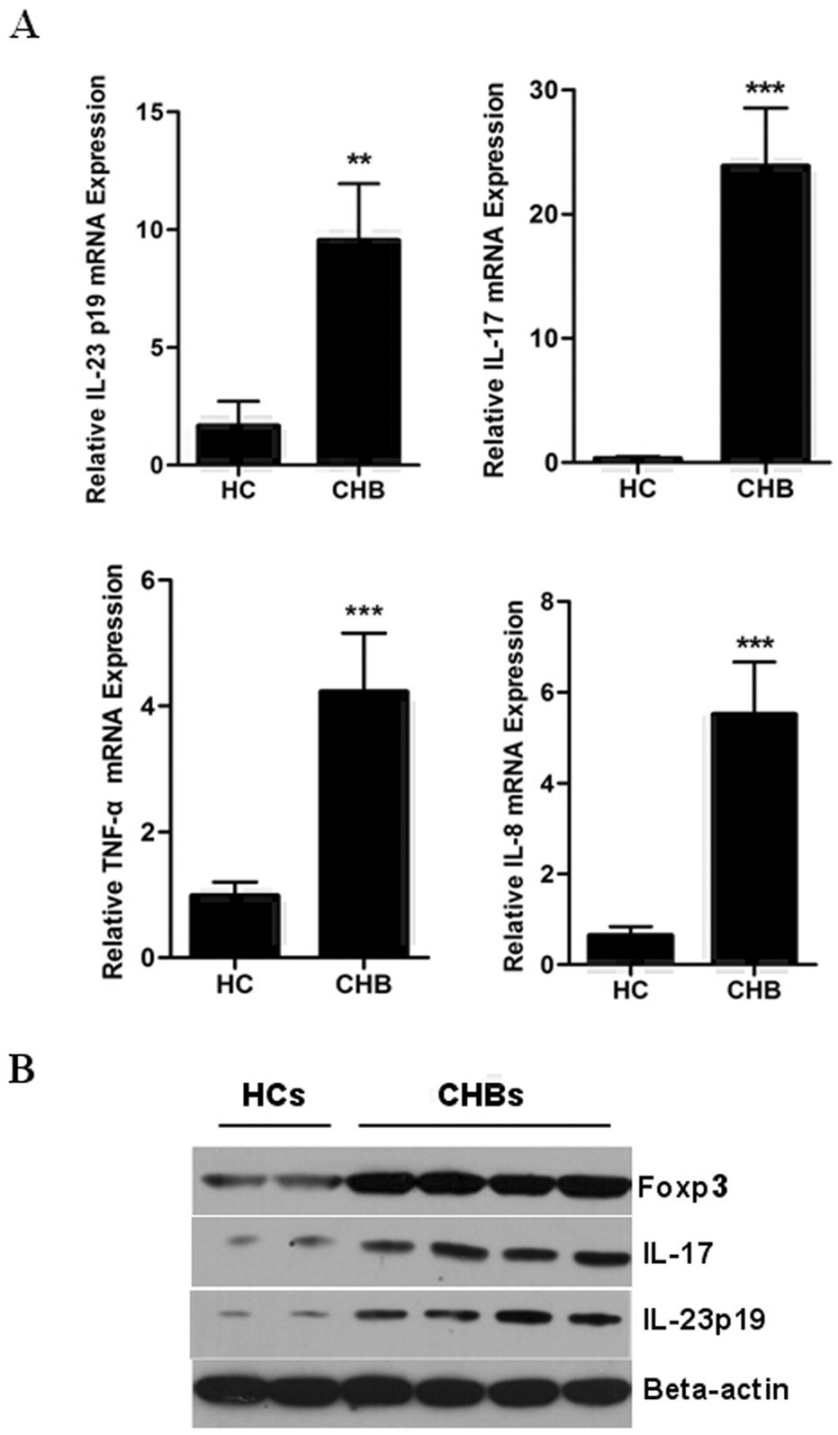
**HBV-infected liver tissues express higher levels of IL-23/IL-17 pathway-related cytokines than non-infected tissues**. Liver biopsy samples from healthy controls and CHB patients were used to detect mRNA level for IL-23, IL-17, TNF-α and IL-8 by qPCR (**A**), and protein expression level of Foxp3, IL-23 and IL-17 by Western-blot assay (**B**). Error bars indicate SD; **, *P *< 0.01 *vs *HC; ***, *P *< 0.001 *vs *HC.

Inflammatory responses are significant features of hepatitis B pathogenesis. It has been reported that the IL-23/IL-17 pathway plays a critical role in inflammatory diseases [17,18]. Moreover, Foxp3 is closely related to the IL-23/IL-17 signaling pathway [19]. In order to further elucidate the role of Foxp3 in patients with CHB, we detected the expression of IL-23/IL-17 pathway-related cytokines in CHB liver samples. As shown in Figure 4A, the mRNA expression of IL-23, IL-17, TNF-α, and IL-8 was significantly higher in these patients liver tissues than in healthy controls. We then investigated whether the protein expression of the key genes, Foxp3, IL-23 and IL-17 genes, was also enhanced in CHB liver tissues by Western-blot assay. Results showed that the protein expression of these three genes in liver tissue of CHB patients was significantly elevated in comparison with that of HC (Figure 4B), suggesting Treg cells and Th17 cells might play important roles simultaneously in CHB pathogenesis.

### Foxp3 expression is positively correlated with presence of IL-23/IL-17 pathway-related cytokines in liver tissue from CHB patients

**Figure 5:**
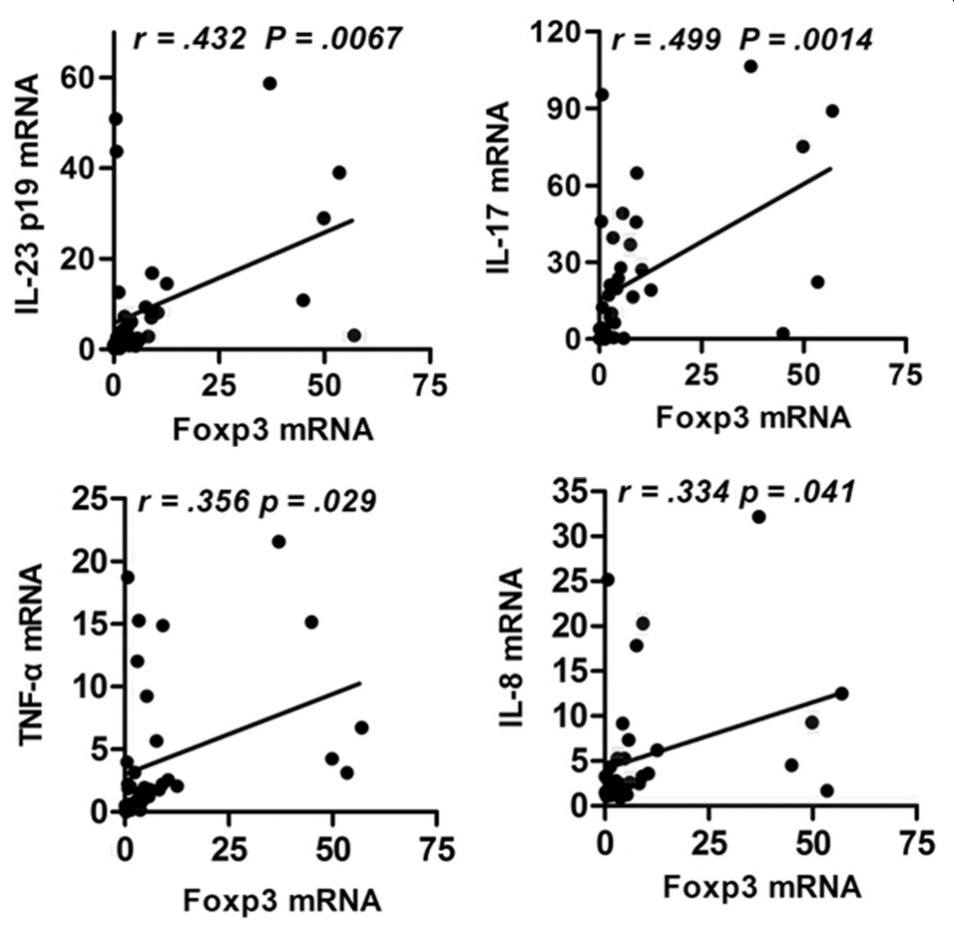
**Foxp3 expression is positively correlated with IL-23/IL-17 pathway in host liver**. Liver biopsy samples from 38 CHB patients were used to detect mRNA for Foxp3, IL-23, IL-17, TNF-α and IL-8 by qPCR, and then the relationship between Foxp3 and IL-23, IL-17, TNF-α or IL-8 was analyzed. *P *< 0.05 was considered significant.

Foxp3 is a potent mediator of dominant self-tolerance and immune homeostasis. Nistala *et al. *[20] demonstrated an inverse relationship between IL-17^+ ^T cells and Foxp3^+ ^Treg cells in joint tissues of children suffering from juvenile arthritis. The fact that Foxp3 and IL-23/IL-17 pathway cytokines were both significantly increased in patients with CHB led us to examine the relationship between these molecules in liver tissues infected with HBV. We found that Foxp3 was significantly correlated with the presence of IL-23 and IL-17 at sites of inflammation (Figure 5).

## Discussion

Immune-mediated liver injury is the key feature of hepatitis B pathogenesis. Recently, much attention has been paid to the importance of Treg cells in chronic HBV infection [21]. In the study described herein, we investigated the expression levels of Foxp3 (the cell-specific transcription factor of Treg cells) and IL-23/IL-17 pathway-related cytokines in HBV-infected liver tissue. We found that Foxp3 and IL-23/IL-17 pathway-related cytokines were significantly higher at sites of inflammation. Moreover, the expression of Foxp3 was closely correlated with the presence of IL-23/IL-17 pathway-related cytokines.

The inflammatory response is necessary to clearance of pathogenic agents, but a fine balance must be maintained so that chronic inflammation does not damage healthy tissues. Treg cells that express Foxp3 play a key role in regulating this delicate equilibrium [22]. Several studies of HBV infection have found that Treg cells are functionally involved in the hepatic immunological responses [6-10]. Likewise, we also observed Foxp3 expression in CD4^+ ^T cells, and not in CD8^+ ^T cells. Moreover, the expression of Foxp3 was significantly increased and in liver tissue of CHB patients. Moreover the mRNA level of Foxp3 at sites of hepatic inflammation was closely correlated with levels of HBV DNA copies and HBsAg. These results indicated that Foxp3 expression was likely to support established residence of HBV.

Recently, clinical investigations have shown that the IL-23/IL-17 signaling pathway contributes to the inflammatory reaction of Crohn's disease, psoriasis and systemic lupus erythematosus [18,23,24]. IL-17 is known as the immune-modulatory factor of Th17 cells, while IL-23 functions to elicit the differentiation and mediate the function of Th17 cells [25]. Several groups have reported that the IL-17 pathway was involved in human liver disease [13,14,26,27]. Our results evidenced that the activity of the IL-23/IL-17 pathway was significantly elevated in liver tissues of patients infected by hepatitis B virus, as compared to that in healthy controls. These results indicated that the IL-23/IL-17 pathway, rather than IL-17 alone, is involved in the pathological process of hepatitis B infection.

Th17 cells and Treg cells both arise from the naive CD4^+ ^T cell population and share reciprocal development pathways. But, they have opposite immunomodulatory effects, and the balance between them controls inflammation and autoimmune diseases [28]. Many studies have identified an imbalance in Th17 cells and Treg cells that occurs in conjunction with disease [6,29-31]. In the present study, we found that expression of Foxp3 and IL-23/IL-17 pathway-related cytokines were increased in inflamed hepatic tissues in CHB patients. We also found that Foxp3 expression was significantly correlated with levels of IL-23/IL-17 pathway-related cytokines in the livers of these CHB patients (Figure 5). Several studies have demonstrated that the development of Th17 cells and Treg cells may be interrelated, as they are both dependent on TGF-β [32]. Yet, they appear to be reciprocally influenced by various cytokines, such as IL-6, IL-1, as well as IL-2 and the vitamin A metabolite all-*trans*-retinoic acid [33-35]. TGF-β is central to the development and function of Th17 and Treg cells. On one hand, TGF-β induces the expression of FoxP3 [36]; on the other hand, TGF-β has been shown to be absolutely required for Th17 differentiation in a serum-free system using cord blood-derived CD4^+ ^T cells [37]. A recent study also showed that Tregs themselves may differentiate into IL-17-producing cells, particularly when exposed to exogenous IL-1β, IL-23 or IL-21 [38,39]. In psoriasis patients, Zhang *et al*. found that Th17 cells were positively correlated with the presence of Treg cells, both in the circulation and in skin tissue lesions [40]. Based on these data, we propose that Tregs may act to promote the pro-inflammatory cytokines, such as IL-17 and IL-23, at certain time points during the course of HBV infection. Considering that the inflammatory response is crucial for clearance of pathogenic microorganisms, it is reasonable that the occurrence of pro-inflammatory immune cells, such as Th17, will be accompanied by inhibitory Tregs cells. In this way, the significant correlation between Treg cells and Th17 cells in the liver of CHB patients is fitting.

## Conclusions

We have demonstrated that Foxp3 and IL-23/IL-17 pathway-related cytokines were both highly expressed in inflamed liver tissues infected by hepatitis B virus. Moreover, the expression of Foxp3 was significantly correlated with that of IL-23/IL-17 pathway-related cytokine genes in the infected liver. These findings support the hypothesis that proinflammatory Th17 (IL-23/IL-17) cells in HBV-infected liver is accompanied by host inflammation-inhibitory machinery, such as Treg (Foxp3) cells, in order to maintain balance between promotion and inhibition of the immune response. Our results also suggested that the activity of the proinflammatory IL-23/IL-17 signaling pathway is not been efficiently controlled by the host inhibitory machinery, in this case the Treg (Foxp3) cells; the constitutive activation of the IL-23/IL-17 signaling pathway may partially explain the HBV persistence that occurs in the chronic hepatitis B state. Future studies will address the precise *in vivo *mechanism of the reciprocal regulation between Foxp3 and the IL-23/IL-17 pathway, and will contribute to development of effective therapies to clear HBV from CHB patients.

## List of Abbreviations Used

HBV: hepatitis B virus; CHB: chronic hepatitis B; Treg: regulatory T cell; Foxp3: forkhead box P3; NK: natural killer cells; NKT: natural killer T cells; DC: dendritic cells; IL: interleukin; Th17: interleukin-17-producing CD4^+ ^T cells; HCV: hepatitis C virus; HDV: hepatitis D virus; HC: healthy controls; FCM: flow cytometry; PBMC: peripheral blood mononuclear cells; BSA: bovine serum albumin; PBS: phosphate buffered saline; DAPI: diaminidophenylindol; ACLF: acute-on-chronic liver failure; SD: standard deviation; HBsAg: hepatitis B surface antigen; ALT: alanine aminotransferase; HBcAg: hepatitis B core Ag; HBeAg: hepatitis B e Ag.

## Declaration

There are no potential conflicts (financial, professional, or personal) that exist for any of the authors in regards to this manuscript.

## Authors' contributions

QW carried out the FCM and quantitative PCR assays. YZ carried out the immunoassays and virological assessmen. ZH performed the statistical analysis. YT participated in the FCM analysis. JZ contributed to the preparation of blood and liver tissue samples from healthy and HBV-infected volunteers. YW and BN conceived of the study, and participated in its design and coordination and drafted the manuscript equally. All authors read and approved the final manuscript.
